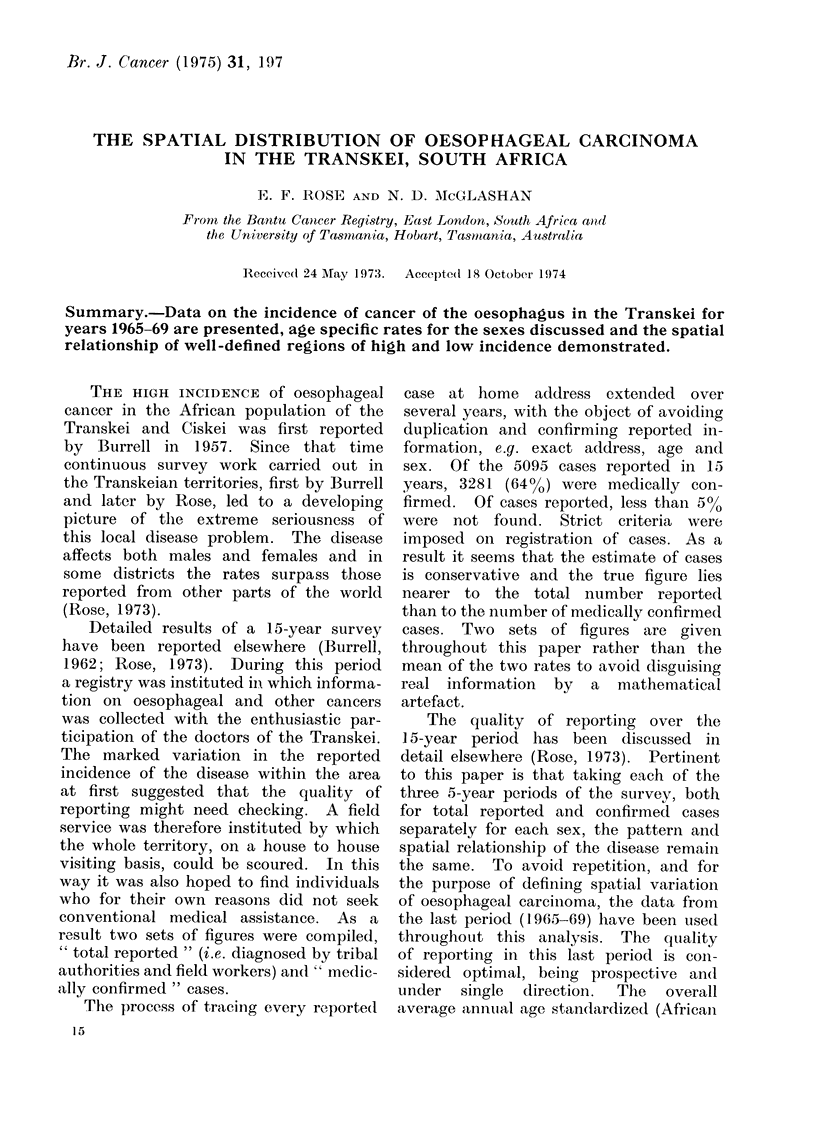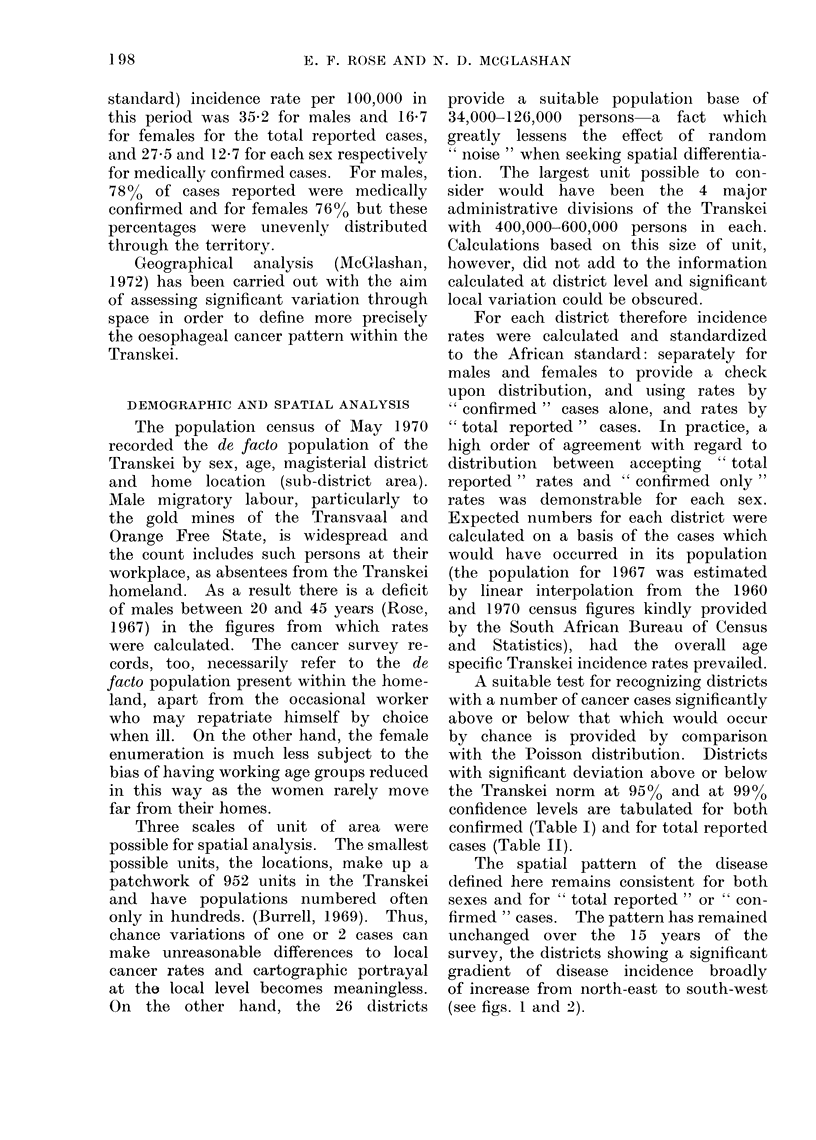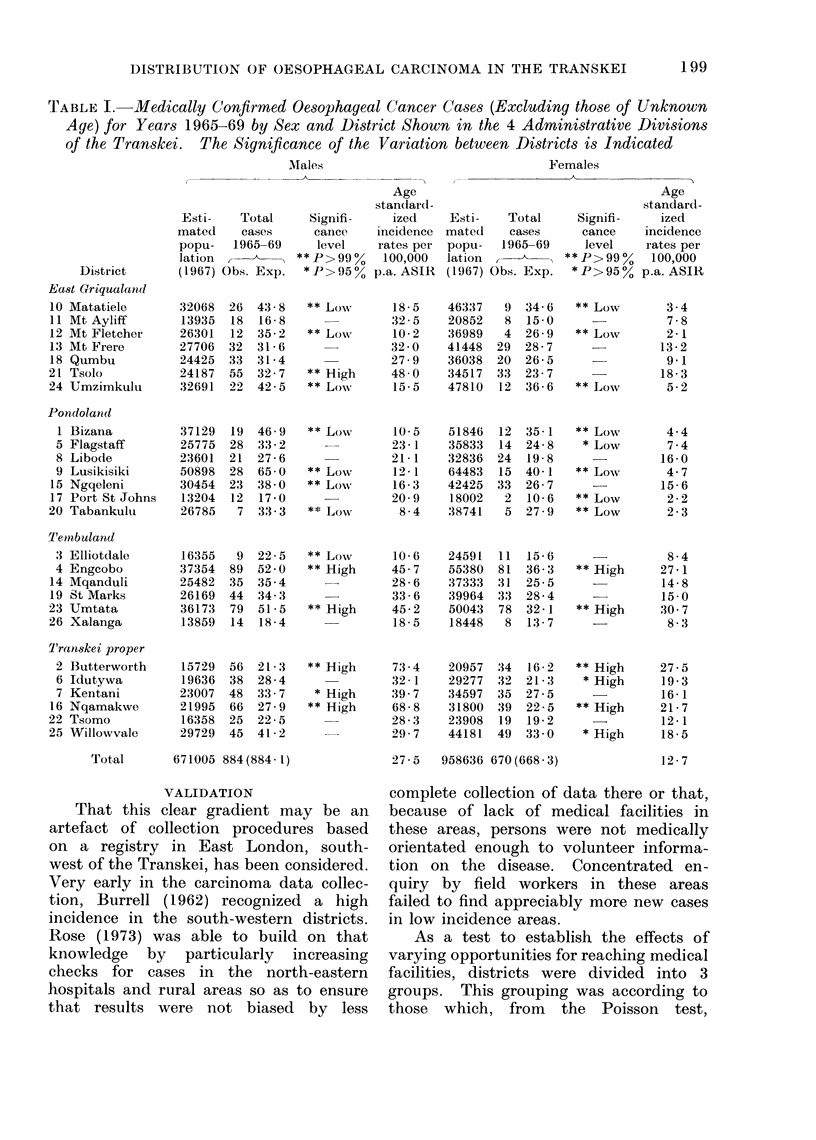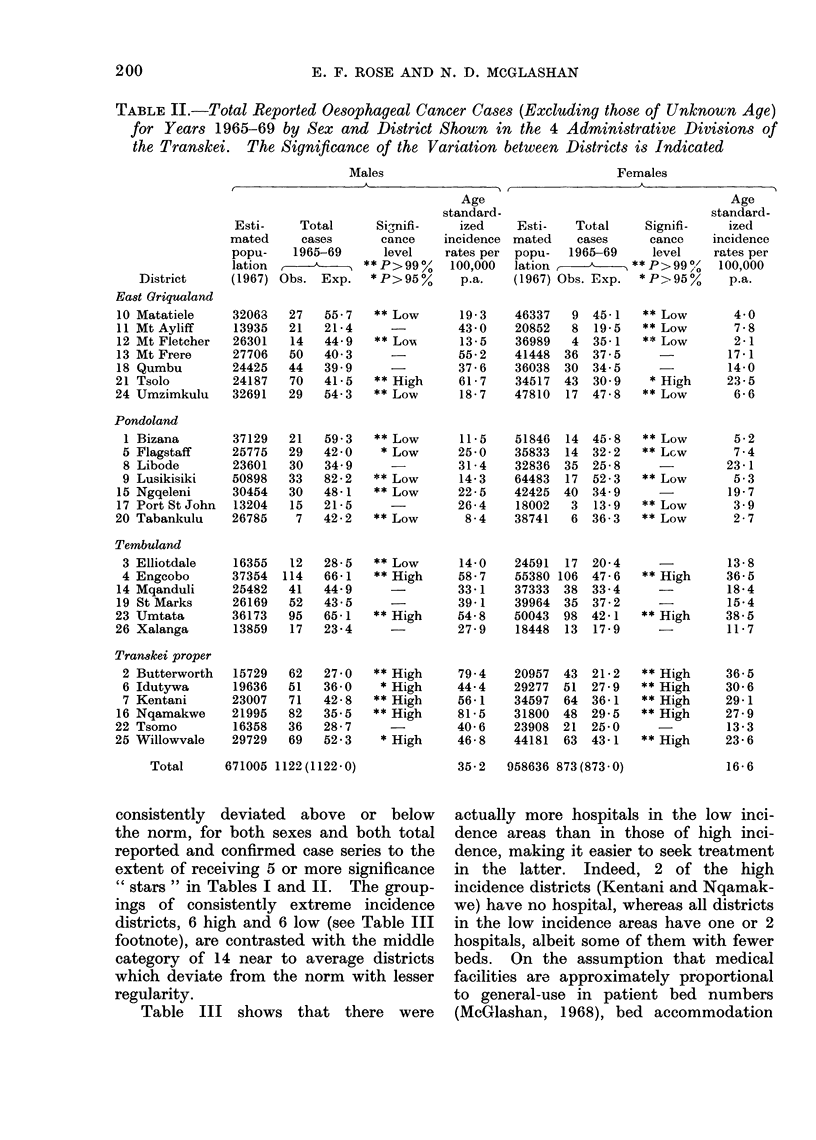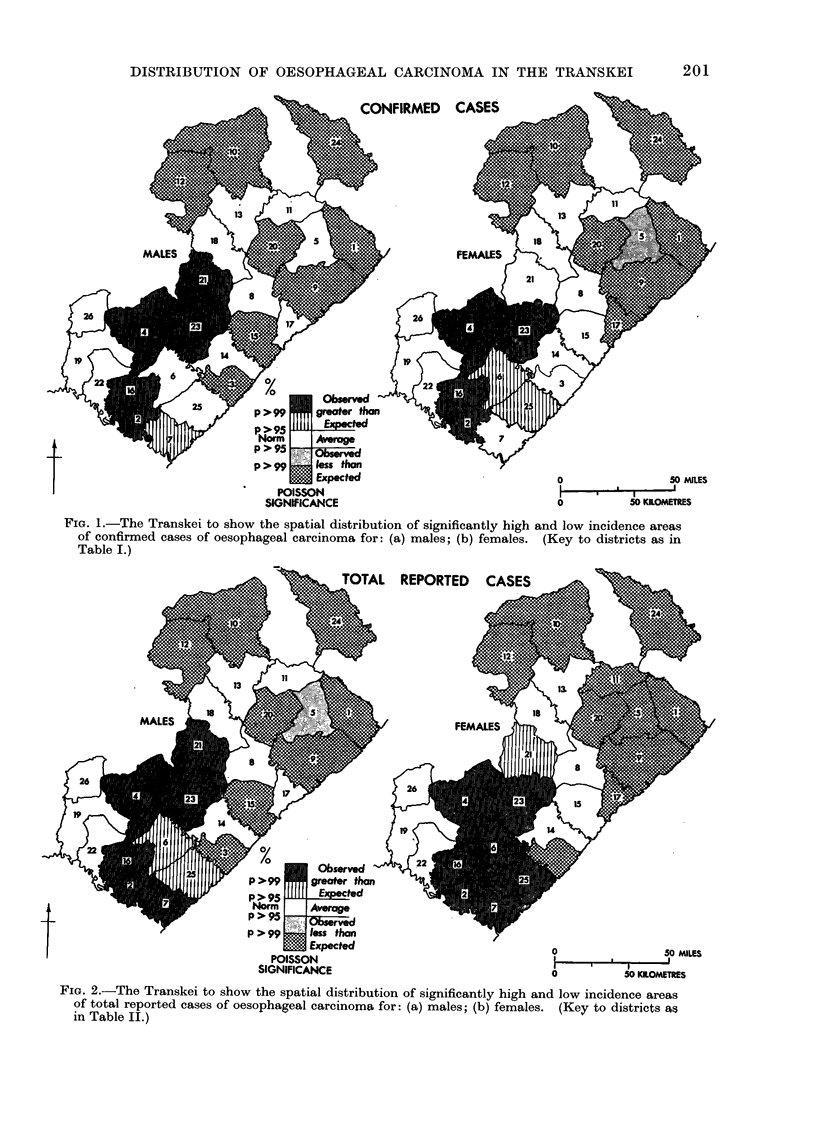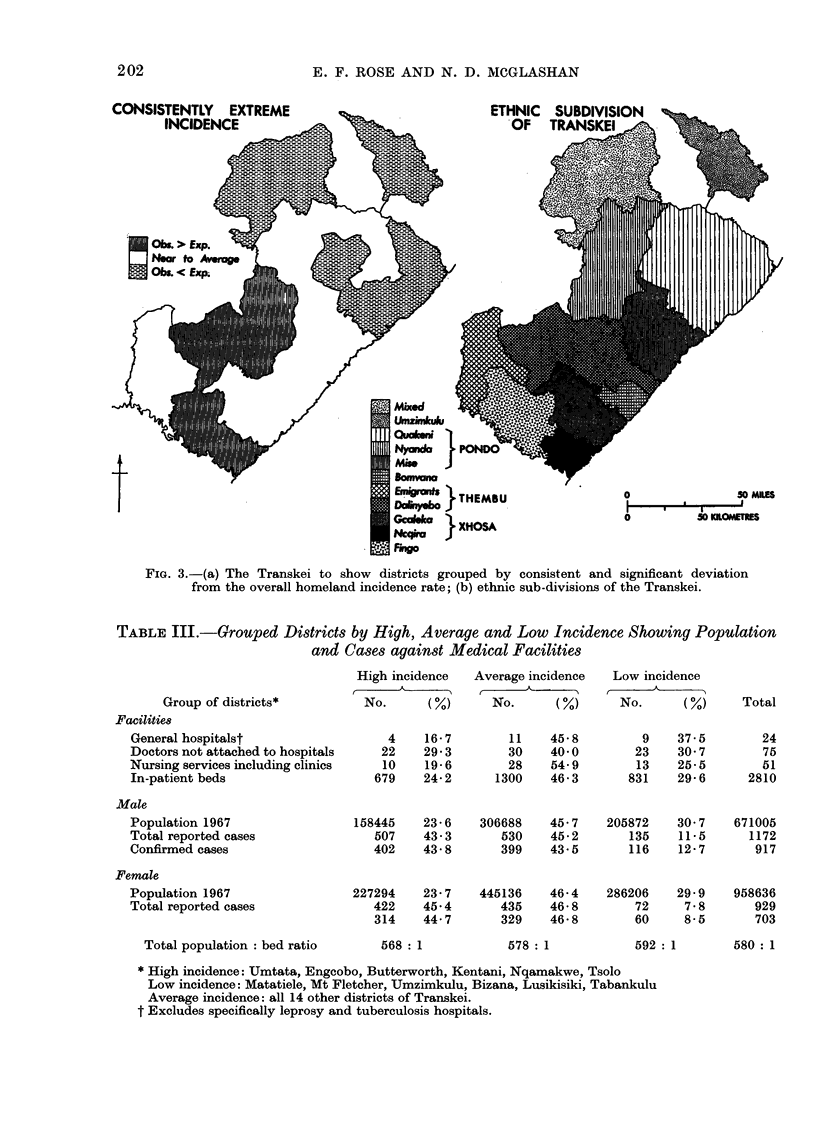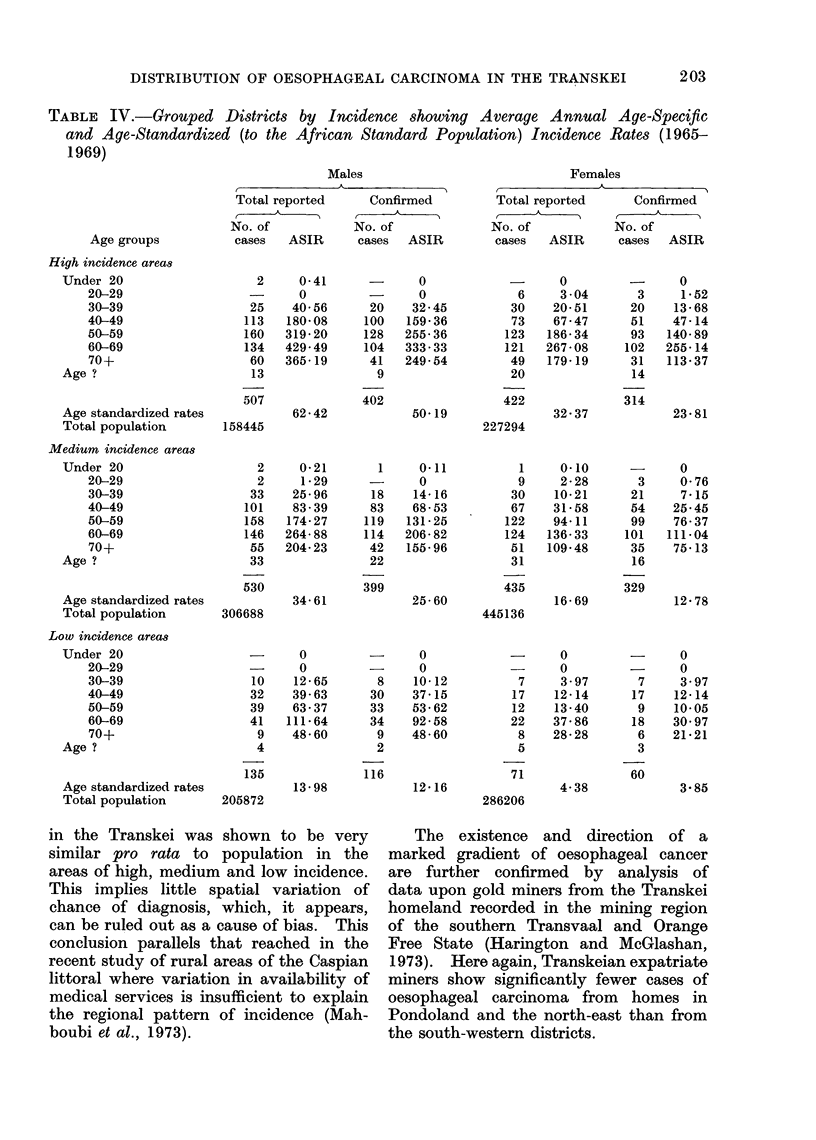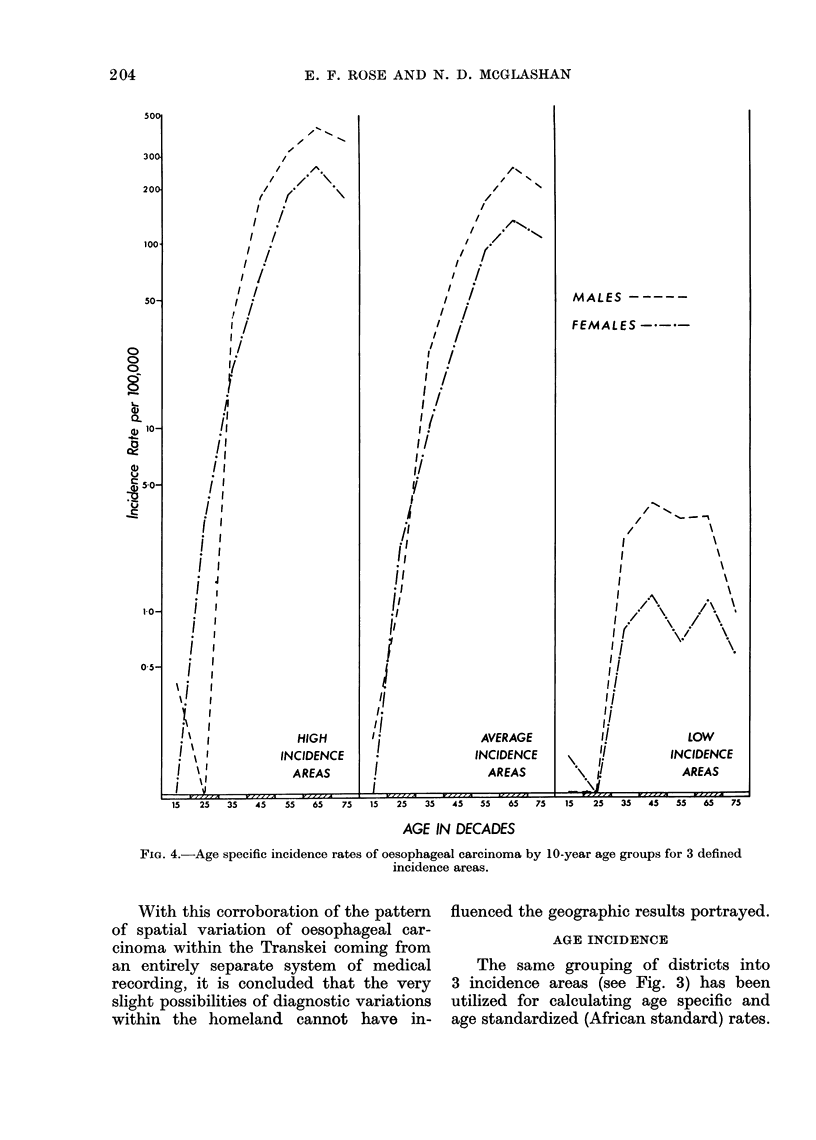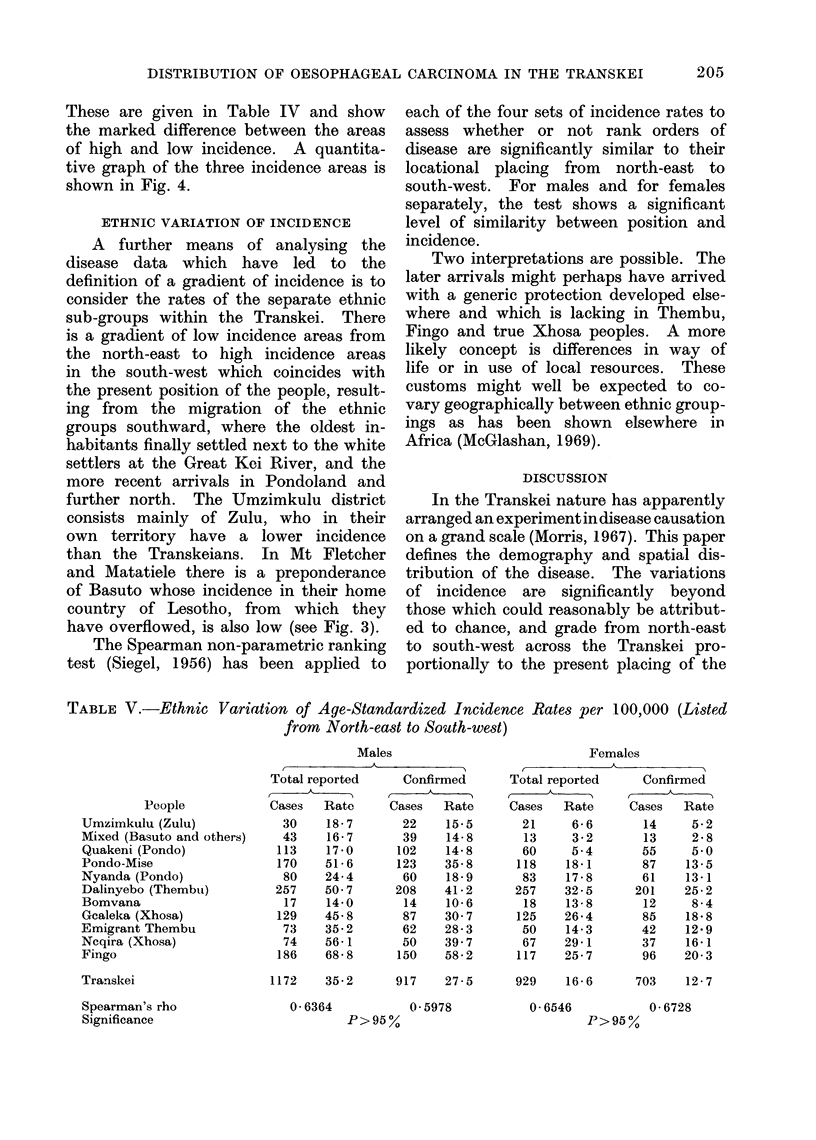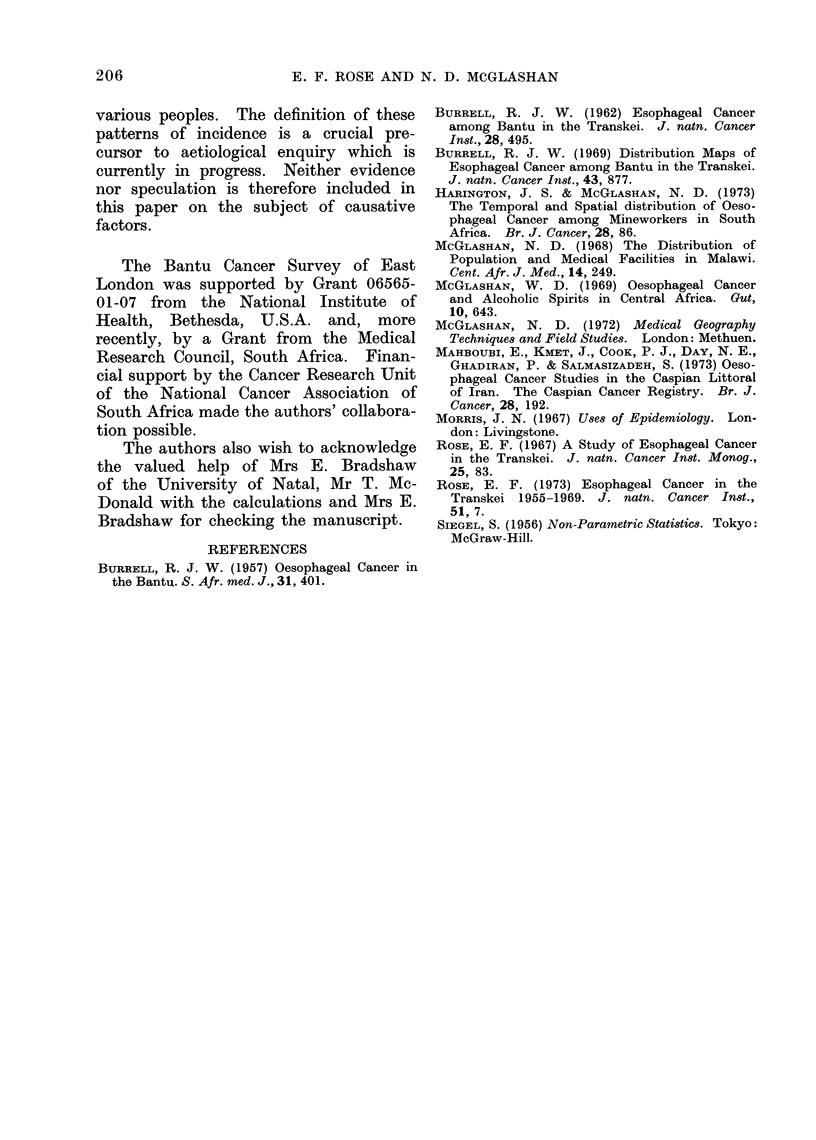# The spatial distribution of oesophageal carcinoma in the Transkei, South Africa.

**DOI:** 10.1038/bjc.1975.26

**Published:** 1975-02

**Authors:** E. F. Rose, N. D. McGlashan

## Abstract

Data on the incidence of cancer of the oesophagus in the Transkei for years 1965-69 are presented, age specific rates for the sexes discussed and the spatial relationship of well-defined regions of the high and low incidence demonstrated.


*Br. J. Cancer* (1975) **31**, 197

### THE SPATIAL DISTRIBUTION OF OESOPHAGEAL CARCINOMA IN THE TRANSKEI, SOUTH AFRICA

E. F. ROSE AND N. D. McGLASHAN

*From the Bantu Cancer Registry, East London, South Africa and the University of Tasmania, Hobart, Tasmania, Australia*

Received 24 May 1973. Accepted 18 October 1974

**Summary.—Data on the incidence of cancer of the oesophagus in the Transkei for years 1965–69 are presented, age specific rates for the sexes discussed and the spatial relationship of well-defined regions of high and low incidence demonstrated.**

THE HIGH INCIDENCE of oesophageal cancer in the African population of the Transkei and Ciskei was first reported by Burrell in 1957. Since that time continuous survey work carried out in the Transkeian territories, first by Burrell and later by Rose, led to a developing picture of the extreme seriousness of this local disease problem. The disease affects both males and females and in some districts the rates surpass those reported from other parts of the world (Rose, 1973).

Detailed results of a 15-year survey have been reported elsewhere (Burrell, 1962; Rose, 1973). During this period a registry was instituted in which information on oesophageal and other cancers was collected with the enthusiastic participation of the doctors of the Transkei. The marked variation in the reported incidence of the disease within the area at first suggested that the quality of reporting might need checking. A field service was therefore instituted by which the whole territory, on a house to house visiting basis, could be scoured. In this way it was also hoped to find individuals who for their own reasons did not seek conventional medical assistance. As a result two sets of figures were compiled, " total reported " (*i.e.* diagnosed by tribal authorities and field workers) and " medically confirmed " cases.

The process of tracing every reported case at home address extended over several years, with the object of avoiding duplication and confirming reported information, *e.g.* exact address, age and sex. Of the 5095 cases reported in 15 years, 3281 (64%) were medically confirmed. Of cases reported, less than 5% were not found. Strict criteria were imposed on registration of cases. As a result it seems that the estimate of cases is conservative and the true figure lies nearer to the total number reported than to the number of medically confirmed cases. Two sets of figures are given throughout this paper rather than the mean of the two rates to avoid disguising real information by a mathematical artefact.

The quality of reporting over the 15-year period has been discussed in detail elsewhere (Rose, 1973). Pertinent to this paper is that taking each of the three 5-year periods of the survey, both for total reported and confirmed cases separately for each sex, the pattern and spatial relationship of the disease remain the same. To avoid repetition, and for the purpose of defining spatial variation of oesophageal carcinoma, the data from the last period (1965–69) have been used throughout this analysis. The quality of reporting in this last period is considered optimal, being prospective and under single direction. The overall average annual age standardized (African standard) incidence rate per 100,000 in this period was 35·2 for males and 16·7 for females for the total reported cases, and 27·5 and 12·7 for each sex respectively for medically confirmed cases. For males, 78% of cases reported were medically confirmed and for females 76% but these percentages were unevenly distributed through the territory.

Geographical analysis (McGlashan, 1972) has been carried out with the aim of assessing significant variation through space in order to define more precisely the oesophageal cancer pattern within the Transkei.

### DEMOGRAPHIC AND SPATIAL ANALYSIS

The population census of May 1970 recorded the *de facto* population of the Transkei by sex, age, magisterial district and home location (sub-district area). Male migratory labour, particularly to the gold mines of the Transvaal and Orange Free State, is widespread and the count includes such persons at their workplace, as absentees from the Transkei homeland. As a result there is a deficit of males between 20 and 45 years (Rose, 1967) in the figures from which rates were calculated. The cancer survey records, too, necessarily refer to the *de facto* population present within the homeland, apart from the occasional worker who may repatriate himself by choice when ill. On the other hand, the female enumeration is much less subject to the bias of having working age groups reduced in this way as the women rarely move far from their homes.

Three scales of unit of area were possible for spatial analysis. The smallest possible units, the locations, make up a patchwork of 952 units in the Transkei and have populations numbered often only in hundreds. (Burrell, 1969). Thus, chance variations of one or 2 cases can make unreasonable differences to local cancer rates and cartographic portrayal at the local level becomes meaningless. On the other hand, the 26 districts provide a suitable population base of 34,000–126,000 persons—a fact which greatly lessens the effect of random "noise" when seeking spatial differentiation. The largest unit possible to consider would have been the 4 major administrative divisions of the Transkei with 400,000–600,000 persons in each. Calculations based on this size of unit, however, did not add to the information calculated at district level and significant local variation could be obscured.

For each district therefore incidence rates were calculated and standardized to the African standard: separately for males and females to provide a check upon distribution, and using rates by "confirmed" cases alone, and rates by "total reported" cases. In practice, a high order of agreement with regard to distribution between accepting "total reported" rates and "confirmed only" rates was demonstrable for each sex. Expected numbers for each district were calculated on a basis of the cases which would have occurred in its population (the population for 1967 was estimated by linear interpolation from the 1960 and 1970 census figures kindly provided by the South African Bureau of Census and Statistics), had the overall age specific Transkei incidence rates prevailed.

A suitable test for recognizing districts with a number of cancer cases significantly above or below that which would occur by chance is provided by comparison with the Poisson distribution. Districts with significant deviation above or below the Transkei norm at 95% and at 99% confidence levels are tabulated for both confirmed (Table I) and for total reported cases (Table II).

The spatial pattern of the disease defined here remains consistent for both sexes and for "total reported" or "confirmed" cases. The pattern has remained unchanged over the 15 years of the survey, the districts showing a significant gradient of disease incidence broadly of increase from north-east to south-west (see figs. 1 and 2).

TABLE I.—*Medically Confirmed Oesophageal Cancer Cases (Excluding those of Unknown Age) for Years 1965–69 by Sex and District Shown in the 4 Administrative Divisions of the Transkei. The Significance of the Variation between Districts is Indicated*

| | Males | | | | | Females | | | | |
|---|---|---|---|---|---|---|---|---|---|---|
| District | Estimated population (1967) | Total cases 1965–69 Obs. | Exp. | Significance level **$P>99\%$ *$P>95\%$ | Age standardized incidence rates per 100,000 p.a. ASIR | Estimated population (1967) | Total cases 1965–69 Obs. | Exp. | Significance level **$P>99\%$ *$P>95\%$ | Age standardized incidence rates per 100,000 p.a. ASIR |
| *East Griqualand* | | | | | | | | | | |
| 10 Matatiele | 32068 | 26 | 43·8 | ** Low | 18·5 | 46337 | 9 | 34·6 | ** Low | 3·4 |
| 11 Mt Ayliff | 13935 | 18 | 16·8 | — | 32·5 | 20852 | 8 | 15·0 | — | 7·8 |
| 12 Mt Fletcher | 26301 | 12 | 35·2 | ** Low | 10·2 | 36989 | 4 | 26·9 | ** Low | 2·1 |
| 13 Mt Frere | 27706 | 32 | 31·6 | — | 32·0 | 41448 | 29 | 28·7 | — | 13·2 |
| 18 Qumbu | 24425 | 33 | 31·4 | — | 27·9 | 36038 | 20 | 26·5 | — | 9·1 |
| 21 Tsolo | 24187 | 55 | 32·7 | ** High | 48·0 | 34517 | 33 | 23·7 | — | 18·3 |
| 24 Umzimkulu | 32691 | 22 | 42·5 | ** Low | 15·5 | 47810 | 12 | 36·6 | ** Low | 5·2 |
| *Pondoland* | | | | | | | | | | |
| 1 Bizana | 37129 | 19 | 46·9 | ** Low | 10·5 | 51846 | 12 | 35·1 | ** Low | 4·4 |
| 5 Flagstaff | 25775 | 28 | 33·2 | — | 23·1 | 35833 | 14 | 24·8 | * Low | 7·4 |
| 8 Libode | 23601 | 21 | 27·6 | — | 21·1 | 32836 | 24 | 19·8 | — | 16·0 |
| 9 Lusikisiki | 50898 | 28 | 65·0 | ** Low | 12·1 | 64483 | 15 | 40·1 | ** Low | 4·7 |
| 15 Ngqeleni | 30454 | 23 | 38·0 | ** Low | 16·3 | 42425 | 33 | 26·7 | — | 15·6 |
| 17 Port St Johns | 13204 | 12 | 17·0 | — | 20·9 | 18002 | 2 | 10·6 | ** Low | 2·2 |
| 20 Tabankulu | 26785 | 7 | 33·3 | ** Low | 8·4 | 38741 | 5 | 27·9 | ** Low | 2·3 |
| *Tembuland* | | | | | | | | | | |
| 3 Elliotdale | 16355 | 9 | 22·5 | ** Low | 10·6 | 24591 | 11 | 15·6 | — | 8·4 |
| 4 Engcobo | 37354 | 89 | 52·0 | ** High | 45·7 | 55380 | 81 | 36·3 | ** High | 27·1 |
| 14 Mqanduli | 25482 | 35 | 35·4 | — | 28·6 | 37333 | 31 | 25·5 | — | 14·8 |
| 19 St Marks | 26169 | 44 | 34·3 | — | 33·6 | 39964 | 33 | 28·4 | — | 15·0 |
| 23 Umtata | 36173 | 79 | 51·5 | ** High | 45·2 | 50043 | 78 | 32·1 | ** High | 30·7 |
| 26 Xalanga | 13859 | 14 | 18·4 | — | 18·5 | 18448 | 8 | 13·7 | — | 8·3 |
| *Transkei proper* | | | | | | | | | | |
| 2 Butterworth | 15729 | 56 | 21·3 | ** High | 73·4 | 20957 | 34 | 16·2 | ** High | 27·5 |
| 6 Idutywa | 19636 | 38 | 28·4 | — | 32·1 | 29277 | 32 | 21·3 | * High | 19·3 |
| 7 Kentani | 23007 | 48 | 33·7 | * High | 39·7 | 34597 | 35 | 27·5 | — | 16·1 |
| 16 Nqamakwe | 21995 | 66 | 27·9 | ** High | 68·8 | 31800 | 39 | 22·5 | ** High | 21·7 |
| 22 Tsomo | 16358 | 25 | 22·5 | — | 28·3 | 23908 | 19 | 19·2 | — | 12·1 |
| 25 Willowvale | 29729 | 45 | 41·2 | — | 29·7 | 44181 | 49 | 33·0 | * High | 18·5 |
| Total | 671005 | 884 | (884·1) | | 27·5 | 958636 | 670 | (668·3) | | 12·7 |

VALIDATION

That this clear gradient may be an artefact of collection procedures based on a registry in East London, south-west of the Transkei, has been considered. Very early in the carcinoma data collection, Burrell (1962) recognized a high incidence in the south-western districts. Rose (1973) was able to build on that knowledge by particularly increasing checks for cases in the north-eastern hospitals and rural areas so as to ensure that results were not biased by less complete collection of data there or that, because of lack of medical facilities in these areas, persons were not medically orientated enough to volunteer information on the disease. Concentrated enquiry by field workers in these areas failed to find appreciably more new cases in low incidence areas.

As a test to establish the effects of varying opportunities for reaching medical facilities, districts were divided into 3 groups. This grouping was according to those which, from the Poisson test,

TABLE II.—*Total Reported Oesophageal Cancer Cases (Excluding those of Unknown Age) for Years 1965–69 by Sex and District Shown in the 4 Administrative Divisions of the Transkei. The Significance of the Variation between Districts is Indicated*

| | Males | | | | | Females | | | | |
|---|---|---|---|---|---|---|---|---|---|---|
| District | Estimated population (1967) | Total cases 1965–69 Obs. | Exp. | Significance level ** $P>99\%$ * $P>95\%$ | Age standardized incidence rates per 100,000 p.a. | Estimated population (1967) | Total cases 1965–69 Obs. | Exp. | Significance level ** $P>99\%$ * $P>95\%$ | Age standardized incidence rates per 100,000 p.a. |
| *East Griqualand* | | | | | | | | | | |
| 10 Matatiele | 32063 | 27 | 55·7 | ** Low | 19·3 | 46337 | 9 | 45·1 | ** Low | 4·0 |
| 11 Mt Ayliff | 13935 | 21 | 21·4 | — | 43·0 | 20852 | 8 | 19·5 | ** Low | 7·8 |
| 12 Mt Fletcher | 26301 | 14 | 44·9 | ** Low | 13·5 | 36989 | 4 | 35·1 | ** Low | 2·1 |
| 13 Mt Frere | 27706 | 50 | 40·3 | — | 55·2 | 41448 | 36 | 37·5 | — | 17·1 |
| 18 Qumbu | 24425 | 44 | 39·9 | — | 37·6 | 36038 | 30 | 34·5 | — | 14·0 |
| 21 Tsolo | 24187 | 70 | 41·5 | ** High | 61·7 | 34517 | 43 | 30·9 | * High | 23·5 |
| 24 Umzimkulu | 32691 | 29 | 54·3 | ** Low | 18·7 | 47810 | 17 | 47·8 | ** Low | 6·6 |
| *Pondoland* | | | | | | | | | | |
| 1 Bizana | 37129 | 21 | 59·3 | ** Low | 11·5 | 51846 | 14 | 45·8 | ** Low | 5·2 |
| 5 Flagstaff | 25775 | 29 | 42·0 | * Low | 25·0 | 35833 | 14 | 32·2 | ** Lcw | 7·4 |
| 8 Libode | 23601 | 30 | 34·9 | — | 31·4 | 32836 | 35 | 25·8 | — | 23·1 |
| 9 Lusikisiki | 50898 | 33 | 82·2 | ** Low | 14·3 | 64483 | 17 | 52·3 | ** Low | 5·3 |
| 15 Ngqeleni | 30454 | 30 | 48·1 | ** Low | 22·5 | 42425 | 40 | 34·9 | — | 19·7 |
| 17 Port St John | 13204 | 15 | 21·5 | — | 26·4 | 18002 | 3 | 13·9 | ** Low | 3·9 |
| 20 Tabankulu | 26785 | 7 | 42·2 | ** Low | 8·4 | 38741 | 6 | 36·3 | ** Low | 2·7 |
| *Tembuland* | | | | | | | | | | |
| 3 Elliotdale | 16355 | 12 | 28·5 | ** Low | 14·0 | 24591 | 17 | 20·4 | — | 13·8 |
| 4 Engcobo | 37354 | 114 | 66·1 | ** High | 58·7 | 55380 | 106 | 47·6 | ** High | 36·5 |
| 14 Mqanduli | 25482 | 41 | 44·9 | — | 33·1 | 37333 | 38 | 33·4 | — | 18·4 |
| 19 St Marks | 26169 | 52 | 43·5 | — | 39·1 | 39964 | 35 | 37·2 | — | 15·4 |
| 23 Umtata | 36173 | 95 | 65·1 | ** High | 54·8 | 50043 | 98 | 42·1 | ** High | 38·5 |
| 26 Xalanga | 13859 | 17 | 23·4 | — | 27·9 | 18448 | 13 | 17·9 | — | 11·7 |
| *Transkei proper* | | | | | | | | | | |
| 2 Butterworth | 15729 | 62 | 27·0 | ** High | 79·4 | 20957 | 43 | 21·2 | ** High | 36·5 |
| 6 Idutywa | 19636 | 51 | 36·0 | * High | 44·4 | 29277 | 51 | 27·9 | ** High | 30·6 |
| 7 Kentani | 23007 | 71 | 42·8 | ** High | 56·1 | 34597 | 64 | 36·1 | ** High | 29·1 |
| 16 Nqamakwe | 21995 | 82 | 35·5 | ** High | 81·5 | 31800 | 48 | 29·5 | ** High | 27·9 |
| 22 Tsomo | 16358 | 36 | 28·7 | — | 40·6 | 23908 | 21 | 25·0 | — | 13·3 |
| 25 Willowvale | 29729 | 69 | 52·3 | * High | 46·8 | 44181 | 63 | 43·1 | ** High | 23·6 |
| Total | 671005 | 1122 | (1122·0) | | 35·2 | 958636 | 873 | (873·0) | | 16·6 |

consistently deviated above or below the norm, for both sexes and both total reported and confirmed case series to the extent of receiving 5 or more significance " stars " in Tables I and II. The groupings of consistently extreme incidence districts, 6 high and 6 low (see Table III footnote), are contrasted with the middle category of 14 near to average districts which deviate from the norm with lesser regularity.

Table III shows that there were actually more hospitals in the low incidence areas than in those of high incidence, making it easier to seek treatment in the latter. Indeed, 2 of the high incidence districts (Kentani and Nqamakwe) have no hospital, whereas all districts in the low incidence areas have one or 2 hospitals, albeit some of them with fewer beds. On the assumption that medical facilities are approximately proportional to general-use in patient bed numbers (McGlashan, 1968), bed accommodation

FIG. 1.—The Transkei to show the spatial distribution of significantly high and low incidence areas of confirmed cases of oesophageal carcinoma for: (a) males; (b) females. (Key to districts as in Table I.)

FIG. 2.—The Transkei to show the spatial distribution of significantly high and low incidence areas of total reported cases of oesophageal carcinoma for: (a) males; (b) females. (Key to districts as in Table II.)

FIG. 3.—(a) The Transkei to show districts grouped by consistent and significant deviation from the overall homeland incidence rate; (b) ethnic sub-divisions of the Transkei.

TABLE III.—*Grouped Districts by High, Average and Low Incidence Showing Population and Cases against Medical Facilities*

| | High incidence | | Average incidence | | Low incidence | | |
|---|---|---|---|---|---|---|---|
| Group of districts* | No. | (%) | No. | (%) | No. | (%) | Total |
| *Facilities* | | | | | | | |
| General hospitals† | 4 | 16·7 | 11 | 45·8 | 9 | 37·5 | 24 |
| Doctors not attached to hospitals | 22 | 29·3 | 30 | 40·0 | 23 | 30·7 | 75 |
| Nursing services including clinics | 10 | 19·6 | 28 | 54·9 | 13 | 25·5 | 51 |
| In-patient beds | 679 | 24·2 | 1300 | 46·3 | 831 | 29·6 | 2810 |
| *Male* | | | | | | | |
| Population 1967 | 158445 | 23·6 | 306688 | 45·7 | 205872 | 30·7 | 671005 |
| Total reported cases | 507 | 43·3 | 530 | 45·2 | 135 | 11·5 | 1172 |
| Confirmed cases | 402 | 43·8 | 399 | 43·5 | 116 | 12·7 | 917 |
| *Female* | | | | | | | |
| Population 1967 | 227294 | 23·7 | 445136 | 46·4 | 286206 | 29·9 | 958636 |
| Total reported cases | 422 | 45·4 | 435 | 46·8 | 72 | 7·8 | 929 |
| | 314 | 44·7 | 329 | 46·8 | 60 | 8·5 | 703 |
| Total population : bed ratio | 568 : 1 | | 578 : 1 | | 592 : 1 | | 580 : 1 |

* High incidence: Umtata, Engcobo, Butterworth, Kentani, Nqamakwe, Tsolo
Low incidence: Matatiele, Mt Fletcher, Umzimkulu, Bizana, Lusikisiki, Tabankulu
Average incidence: all 14 other districts of Transkei.
† Excludes specifically leprosy and tuberculosis hospitals.

TABLE IV.—*Grouped Districts by Incidence showing Average Annual Age-Specific and Age-Standardized (to the African Standard Population) Incidence Rates* (1965–1969)

| | Males | | | | Females | | | |
|---|---|---|---|---|---|---|---|---|
| | Total reported | | Confirmed | | Total reported | | Confirmed | |
| Age groups | No. of cases | ASIR | No. of cases | ASIR | No. of cases | ASIR | No. of cases | ASIR |
| *High incidence areas* | | | | | | | | |
| Under 20 | 2 | 0·41 | — | 0 | — | 0 | — | 0 |
| 20–29 | — | 0 | — | 0 | 6 | 3·04 | 3 | 1·52 |
| 30–39 | 25 | 40·56 | 20 | 32·45 | 30 | 20·51 | 20 | 13·68 |
| 40–49 | 113 | 180·08 | 100 | 159·36 | 73 | 67·47 | 51 | 47·14 |
| 50–59 | 160 | 319·20 | 128 | 255·36 | 123 | 186·34 | 93 | 140·89 |
| 60–69 | 134 | 429·49 | 104 | 333·33 | 121 | 267·08 | 102 | 255·14 |
| 70+ | 60 | 365·19 | 41 | 249·54 | 49 | 179·19 | 31 | 113·37 |
| Age ? | 13 | | 9 | | 20 | | 14 | |
| | 507 | | 402 | | 422 | | 314 | |
| Age standardized rates | | 62·42 | | 50·19 | | 32·37 | | 23·81 |
| Total population | 158445 | | | | 227294 | | | |
| *Medium incidence areas* | | | | | | | | |
| Under 20 | 2 | 0·21 | 1 | 0·11 | 1 | 0·10 | — | 0 |
| 20–29 | 2 | 1·29 | — | 0 | 9 | 2·28 | 3 | 0·76 |
| 30–39 | 33 | 25·96 | 18 | 14·16 | 30 | 10·21 | 21 | 7·15 |
| 40–49 | 101 | 83·39 | 83 | 68·53 | 67 | 31·58 | 54 | 25·45 |
| 50–59 | 158 | 174·27 | 119 | 131·25 | 122 | 94·11 | 99 | 76·37 |
| 60–69 | 146 | 264·88 | 114 | 206·82 | 124 | 136·33 | 101 | 111·04 |
| 70+ | 55 | 204·23 | 42 | 155·96 | 51 | 109·48 | 35 | 75·13 |
| Age ? | 33 | | 22 | | 31 | | 16 | |
| | 530 | | 399 | | 435 | | 329 | |
| Age standardized rates | | 34·61 | | 25·60 | | 16·69 | | 12·78 |
| Total population | 306688 | | | | 445136 | | | |
| *Low incidence areas* | | | | | | | | |
| Under 20 | — | 0 | — | 0 | — | 0 | — | 0 |
| 20–29 | — | 0 | — | 0 | — | 0 | — | 0 |
| 30–39 | 10 | 12·65 | 8 | 10·12 | 7 | 3·97 | 7 | 3·97 |
| 40–49 | 32 | 39·63 | 30 | 37·15 | 17 | 12·14 | 17 | 12·14 |
| 50–59 | 39 | 63·37 | 33 | 53·62 | 12 | 13·40 | 9 | 10·05 |
| 60–69 | 41 | 111·64 | 34 | 92·58 | 22 | 37·86 | 18 | 30·97 |
| 70+ | 9 | 48·60 | 9 | 48·60 | 8 | 28·28 | 6 | 21·21 |
| Age ? | 4 | | 2 | | 5 | | 3 | |
| | 135 | | 116 | | 71 | | 60 | |
| Age standardized rates | | 13·98 | | 12·16 | | 4·38 | | 3·85 |
| Total population | 205872 | | | | 286206 | | | |

in the Transkei was shown to be very similar *pro rata* to population in the areas of high, medium and low incidence. This implies little spatial variation of chance of diagnosis, which, it appears, can be ruled out as a cause of bias. This conclusion parallels that reached in the recent study of rural areas of the Caspian littoral where variation in availability of medical services is insufficient to explain the regional pattern of incidence (Mahboubi *et al.*, 1973).

The existence and direction of a marked gradient of oesophageal cancer are further confirmed by analysis of data upon gold miners from the Transkei homeland recorded in the mining region of the southern Transvaal and Orange Free State (Harington and McGlashan, 1973). Here again, Transkeian expatriate miners show significantly fewer cases of oesophageal carcinoma from homes in Pondoland and the north-east than from the south-western districts.

FIG. 4.—Age specific incidence rates of oesophageal carcinoma by 10-year age groups for 3 defined incidence areas.

With this corroboration of the pattern of spatial variation of oesophageal carcinoma within the Transkei coming from an entirely separate system of medical recording, it is concluded that the very slight possibilities of diagnostic variations within the homeland cannot have influenced the geographic results portrayed.

### AGE INCIDENCE

The same grouping of districts into 3 incidence areas (see Fig. 3) has been utilized for calculating age specific and age standardized (African standard) rates.

These are given in Table IV and show the marked difference between the areas of high and low incidence. A quantitative graph of the three incidence areas is shown in Fig. 4.

### ETHNIC VARIATION OF INCIDENCE

A further means of analysing the disease data which have led to the definition of a gradient of incidence is to consider the rates of the separate ethnic sub-groups within the Transkei. There is a gradient of low incidence areas from the north-east to high incidence areas in the south-west which coincides with the present position of the people, resulting from the migration of the ethnic groups southward, where the oldest inhabitants finally settled next to the white settlers at the Great Kei River, and the more recent arrivals in Pondoland and further north. The Umzimkulu district consists mainly of Zulu, who in their own territory have a lower incidence than the Transkeians. In Mt Fletcher and Matatiele there is a preponderance of Basuto whose incidence in their home country of Lesotho, from which they have overflowed, is also low (see Fig. 3).

The Spearman non-parametric ranking test (Siegel, 1956) has been applied to each of the four sets of incidence rates to assess whether or not rank orders of disease are significantly similar to their locational placing from north-east to south-west. For males and for females separately, the test shows a significant level of similarity between position and incidence.

Two interpretations are possible. The later arrivals might perhaps have arrived with a generic protection developed elsewhere and which is lacking in Thembu, Fingo and true Xhosa peoples. A more likely concept is differences in way of life or in use of local resources. These customs might well be expected to covary geographically between ethnic groupings as has been shown elsewhere in Africa (McGlashan, 1969).

### DISCUSSION

In the Transkei nature has apparently arranged an experiment in disease causation on a grand scale (Morris, 1967). This paper defines the demography and spatial distribution of the disease. The variations of incidence are significantly beyond those which could reasonably be attributed to chance, and grade from north-east to south-west across the Transkei proportionally to the present placing of the

TABLE V.—*Ethnic Variation of Age-Standardized Incidence Rates per* 100,000 (*Listed from North-east to South-west*)

| | Males | | | | Females | | | |
|---|---|---|---|---|---|---|---|---|
| | Total reported | | Confirmed | | Total reported | | Confirmed | |
| People | Cases | Rate | Cases | Rate | Cases | Rate | Cases | Rate |
| Umzimkulu (Zulu) | 30 | 18·7 | 22 | 15·5 | 21 | 6·6 | 14 | 5·2 |
| Mixed (Basuto and others) | 43 | 16·7 | 39 | 14·8 | 13 | 3·2 | 13 | 2·8 |
| Quakeni (Pondo) | 113 | 17·0 | 102 | 14·8 | 60 | 5·4 | 55 | 5·0 |
| Pondo-Mise | 170 | 51·6 | 123 | 35·8 | 118 | 18·1 | 87 | 13·5 |
| Nyanda (Pondo) | 80 | 24·4 | 60 | 18·9 | 83 | 17·8 | 61 | 13·1 |
| Dalinyebo (Thembu) | 257 | 50·7 | 208 | 41·2 | 257 | 32·5 | 201 | 25·2 |
| Bomvana | 17 | 14·0 | 14 | 10·6 | 18 | 13·8 | 12 | 8·4 |
| Gcaleka (Xhosa) | 129 | 45·8 | 87 | 30·7 | 125 | 26·4 | 85 | 18·8 |
| Emigrant Thembu | 73 | 35·2 | 62 | 28·3 | 50 | 14·3 | 42 | 12·9 |
| Ncqira (Xhosa) | 74 | 56·1 | 50 | 39·7 | 67 | 29·1 | 37 | 16·1 |
| Fingo | 186 | 68·8 | 150 | 58·2 | 117 | 25·7 | 96 | 20·3 |
| Transkei | 1172 | 35·2 | 917 | 27·5 | 929 | 16·6 | 703 | 12·7 |
| Spearman's rho | 0·6364 | | 0·5978 | | 0·6546 | | 0·6728 | |
| Significance | | $P > 95\%$ | | | | $P > 95\%$ | | |

various peoples. The definition of these patterns of incidence is a crucial precursor to aetiological enquiry which is currently in progress. Neither evidence nor speculation is therefore included in this paper on the subject of causative factors.

The Bantu Cancer Survey of East London was supported by Grant 06565-01-07 from the National Institute of Health, Bethesda, U.S.A. and, more recently, by a Grant from the Medical Research Council, South Africa. Financial support by the Cancer Research Unit of the National Cancer Association of South Africa made the authors' collaboration possible.

The authors also wish to acknowledge the valued help of Mrs E. Bradshaw of the University of Natal, Mr T. McDonald with the calculations and Mrs E. Bradshaw for checking the manuscript.

### REFERENCES

BURRELL, R. J. W. (1957) Oesophageal Cancer in the Bantu. *S. Afr. med. J.*, **31**, 401.

BURRELL, R. J. W. (1962) Esophageal Cancer among Bantu in the Transkei. *J. natn. Cancer Inst.*, **28**, 495.

BURRELL, R. J. W. (1969) Distribution Maps of Esophageal Cancer among Bantu in the Transkei. *J. natn. Cancer Inst.*, **43**, 877.

HARINGTON, J. S. & MCGLASHAN, N. D. (1973) The Temporal and Spatial distribution of Oesophageal Cancer among Mineworkers in South Africa. *Br. J. Cancer*, **28**, 86.

MCGLASHAN, N. D. (1968) The Distribution of Population and Medical Facilities in Malawi. *Cent. Afr. J. Med.*, **14**, 249.

MCGLASHAN, W. D. (1969) Oesophageal Cancer and Alcoholic Spirits in Central Africa. *Gut*, **10**, 643.

MCGLASHAN, N. D. (1972) *Medical Geography Techniques and Field Studies*. London: Methuen.

MAHBOUBI, E., KMET, J., COOK, P. J., DAY, N. E., GHADIRAN, P. & SALMASIZADEH, S. (1973) Oesophageal Cancer Studies in the Caspian Littoral of Iran. The Caspian Cancer Registry. *Br. J. Cancer*, **28**, 192.

MORRIS, J. N. (1967) *Uses of Epidemiology*. London: Livingstone.

ROSE, E. F. (1967) A Study of Esophageal Cancer in the Transkei. *J. natn. Cancer Inst. Monog.*, **25**, 83.

ROSE, E. F. (1973) Esophageal Cancer in the Transkei 1955–1969. *J. natn. Cancer Inst.*, **51**, 7.

SIEGEL, S. (1956) *Non-Parametric Statistics*. Tokyo: McGraw-Hill.